# Role of Hippo-YAP Signaling in Osseointegration by Regulating Osteogenesis, Angiogenesis, and Osteoimmunology

**DOI:** 10.3389/fcell.2020.00780

**Published:** 2020-08-19

**Authors:** Anqi Zhou, Hui Yu, Jiayi Liu, Jianan Zheng, Yinan Jia, Bingfeng Wu, Lin Xiang

**Affiliations:** ^1^State Key Laboratory of Oral Diseases & National Clinical Research Center for Oral Diseases, West China Hospital of Stomatology, Sichuan University, Chengdu, China; ^2^Department of Oral Implantology, West China Hospital of Stomatology, Sichuan University, Chengdu, China

**Keywords:** osseointegration, Hippo-YAP, osteogenesis, angiogenesis, osteoimmunology, bone remodeling, implants

## Abstract

The social demand for dental implantation is growing at a rapid rate, while dentists are faced with the dilemma of implantation failures associated with unfavorable osseointegration. Clinical-friendly osteogenesis, angiogenesis and osteoimmunology around dental implants play a pivotal role in a desirable osseointegration and it’s increasingly appreciated that Hippo-YAP signaling pathway is implicated in those biological processes both *in vitro* and *in vivo* in a variety of study. In this article we review the multiple effects of Hippo-YAP signaling in osseointegration of dental implants by regulating osteogenesis, angiogenesis and osteoimmunology in peri-implant tissue, as well as highlight prospective future directions of relevant investigation.

## Introduction

With the increasing social burden of growing elderly population, some age-implicated diseases are changing people’s demand for medical services including tooth loss. American Association of Oral and Maxillofacial Surgeons illustrates that 70% adults between 35 and 44 years old have at least one permanent tooth lost and 26% adults lose all by 74 years old, which has significant impacts on general health physically and mentally through direct and indirect mechanisms (Barboza-Solís et al., 2019; Bollman et al., 2020). Therefore, high-quality and efficient treatment to restore the function and esthetics for the cases of tooth loss is in increasing demand right now and facing great challenges. In the past, removable dentures and bridges were used in patients to replace missing teeth. However, over the last few decades dental implant has become very popular and a mainstream treatment for the advantages of high predictability and success rate as well as fewer complications during and after implantation (Shemtov-Yona and Rittel, 2015; Zohrabian et al., 2015). In addition, Howe et al. conducted a meta-analysis of 10-year dental implant survival and it turned out that the survival rate was 96.4% (Howe et al., 2019). However, while the demand for dental implants is growing at a very rapid rate, dentists are faced with the dilemma of dental implant failure associated with peri-implant mucositis, peri-implantitis, esthetic failures, and complete loss of osseointegration in clinical cases (Hickin et al., 2017).

Brånemark originally proposed the concept of osseointegration to describe the direct and stable connection between bone tissue and titanium implants. Zarb proposed a clinical description that it was a clinically asymptomatic fixation of functional-loaded implants (Zarb and Albrektsson, 1991). A desirable osseointegration is the key to a success implantation, which has been an ultimate goal for dentists to achieve. Bai et al. (2018a) attributed most implant failures to insufficient osseointegration between host bone and the surface of implants. In recent years, research on improving osseointegration to gain a higher survival rate of dental implant has become a hot topic in dentistry.

A favorable osseointegration of bone-implant interface is attributed to peri-implant osteogenesis, angiogenesis and osteoimmunology properties. On the one hand, these three factors play their respective important roles in regulating the process of osseointegration. Peri-implant bone osteogenesis is indispensable for stability and function of dental implants, which is regulated by the dynamic balance of osteoblasts, osteoclasts, osteocytes, etc. (Insua et al., 2017; Bai et al., 2018a). Angiogenesis is also an important component of accelerating bone repair since newborn blood vessels provide oxygen and nutrients for bone tissue and create routines for cell migration (Hankenson et al., 2011). As foreign bodies in bone tissue, successful dental implant is strongly dependent on a promising local immune microenvironment and proper osteoimmunomodulation to reach a favorable osseointegration which is dominated by the variety of peri-implant immune cells (Bai et al., 2018b; Wang J. et al., 2018.) On the other hand, the three factors are highly related and interact on each other. Numerous compelling evidences have showed that osteogenesis and angiogenesis are coupled to promote bone regeneration by wild cross-talk via various mediators and signals and both accelerated by favorable osteoimmunology properties to reach a clinical-friendly osseointegration, therefore the concept of osteoimmunology and osteo/angio-genesis overlap to a certain degree (Dohle et al., 2014; Shi et al., 2016, 2017; Okamoto et al., 2017; Bai et al., 2018a; Ma et al., 2018; Trindade et al., 2018; Tsukasaki and Takayanagi, 2019; Brunetti et al., 2020; Gao et al., 2020; Guder et al., 2020). Nevertheless, the specific regulation mechanisms on these three independent but wildly interrelated biological processes remain to be further clarified.

Hippo-YAP, a highly implicated pathway, is known to be involved in regulating organ size, tissue regeneration and cancer development. Hippo signaling senses and responds to upstream cell biomechanical cues including cell contacts, cell polarity and other biomechanical signals. MST1/2 and SAV1 are phosphorylated and activate the complex of LATS1/2 and MOB1A/B, thus activating downstream reaction (Xiang et al., 2018). Negatively modulated by Hippo signaling, Yes-associated protein (YAP) is a key downstream effector and regulates various cell properties such as controlling cell proliferation and fate by influencing gene expression with transcriptional enhancer associated domain transcription factors (TEADs), the main transcriptional factors interact with YAP.

## Osteogenesis, Angiogenesis and Osteoimmunology and Their Effects on Osseointegration (Figure 1)

### Osteoimmunology

The concept of osteoimmunology was first established by Arror et al., emphasizing the interaction between skeletal and immune system (Arron and Choi, 2000; Tsukasaki and Takayanagi, 2019). On the one hand, cells of skeletal system are involved in immune regulation by secreting key cytokines in bone marrow microenvironment, where immune cells and their progenitors are harbored and nourished initially (Walsh et al., 2018; Tsukasaki and Takayanagi, 2019). On the other hand, the abnormal activation of immune cells may affect osteogenesis and angiogenesis, contributing to the development of pathological bone damage diseases, such as periodontitis and rheumatoid arthritis, as well as slow bone repair. As the bridge between skeletal and immune system, osteoimmunology plays a significant regulatory role in a variety of essential biological processes, including the osseointegration procedure of implants.

**FIGURE 1:**
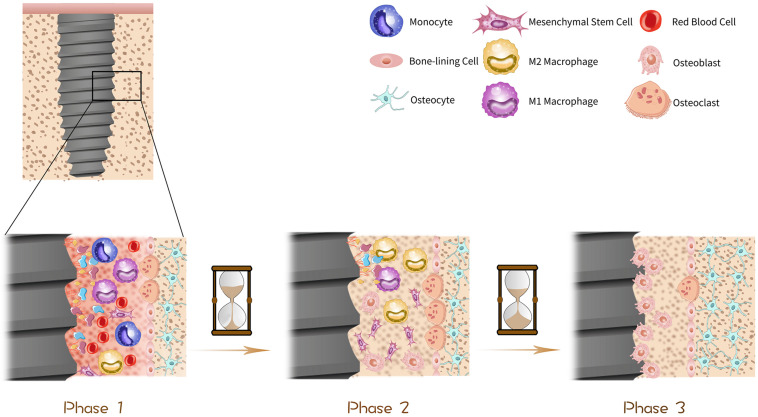
Procedure of osseointegration. The histological change of dental implants in bone can be divided into three phases in general. In phase one, as soon as the implantation, the implant surface is surrounded by blood and immediately biomolecules including proteins, lipids and glycoproteins are absorbed to the surface, forming a temporary bioactive layer, with bone marrow cells scattering around. In phase two, about 1 month after the operation, some parts of bone tissue become absorbed due to the excessive pressure exerted on them, which is driven by osteoclasts. Simultaneously, the temporary bioactive layer is absorbed by macrophages and osteogenic cells such as mesenchymal stem cells (MSCs) and osteoblasts are stimulated to migrate to osteogenic area on the surface of implants and start to proliferate and differentiate thus mineralization procedure is initiated. In phase three, about 3 months after the implantation, the implant surface is surrounded by osteoblasts and osteocytes which get mature gradually, thus osseointegration is done progressively over time.

Inflammatory response around dental implants is usually considered a pathological process. In fact, as a process of direct connection between foreign body and bone tissue, osseointegration would initially raise the foreign-body reaction to implants. The foreign-body reaction starts with layer of proteins that come from blood and interstitium immediately forming on the surface of biomaterial after implantation, which activates inflammatory reaction of related cells as a result (Wilson et al., 2005; Singhatanadgit et al., 2019). Researches on enhancing the biocompatibility of implant material have been a hot topic in dentistry, for instance, surface modification (Hamlet and Ivanovski, 2011; Hamlet et al., 2012; Palmquist et al., 2013; Fukuda et al., 2019). as well as changing the design and composition of implants (Cooper et al., 2016; Wang Y. et al., 2019) are generally proposed as available strategies. Additionally, biofilm consisted of various subgingival bacteria may form upon the material at once after implantation, which stimulates excessive inflammatory response provided that microbiome dysbiosis occurs, leading peri-implant mucositis or even peri-implantitis that threatens the stability and survival of dental implants (Wisdom et al., 2019).

**FIGURE 2:**
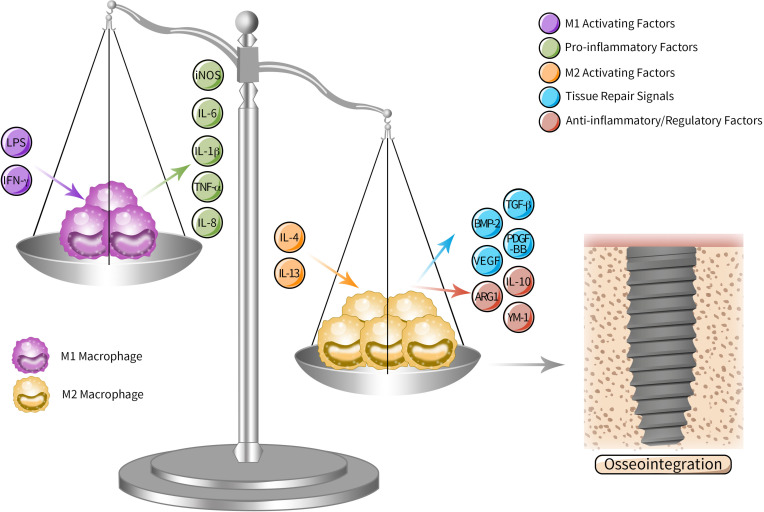
M1/M2 polarization spectrum in osteoimmunology mediated osseointegration. As the key participants in peri-implant osteoimmunology, macrophages can be subdivided into M1 and M2 according to the activation pathway, secretion and function. M1 is the pro-inflammatory type activated by M1 activating factors such as LPS and IFN-γ, inducing excessive fibrosis and osseointegration failure by secreting pro-inflammatory cytokines, while M2 is activated by M2 activating factors such as IL-4 and IL-13, which promote osseointegration through anti-inflammatory and regulatory cytokines. Although *in vivo* macrophages actually display an intermediate state along the polarization spectrum, tipping the polarizing “scale” of macrophage from M1 to M2 leads to influencing microenvironment of inflammation and regeneration around implants and promoting osseointegration.

But our current knowledge of the effects of inflammatory response is that osseointegration is a complicated process relying on a dynamically balanced early inflammatory response of immune cells to implant, especially the response performed by macrophages, the main participants in reacting to biomaterials (Brown et al., 2017; Gibon et al., 2017; Lee and Bance, 2019). According to the activation pathway, secretion and function, macrophages are classified into classically activated macrophages (M1) and alternatively activated macrophages (M2). M1/M2 macrophages lead to opposite reacting process in response to different microenvironment. M1 is described as pro-inflammatory cell type that induces osseointegration failure with a layer of fibrous tissue surrounding the implants. While M2 is the anti-inflammatory/regulatory one (Brown et al., 2017) that attracts cells, proteins and other bioactive substances around implants and hence plays a dominant role in osseointegration. However, *in vivo* the two extreme polarization states hardly exist since in most cases macrophages display both M1 and M2 characteristics phenotypes and exist as an intermediate state along the polarization spectrum (Brown et al., 2017). Although there are more subtypes and advanced classification pattern discussed nowadays, Wang et al. propose that regulating macrophage polarizing along M1 and M2 direction leads to influencing microenvironment of inflammation and regeneration thus coordinating osseointegration (Wang J. et al., 2018). The classic M1/M2 dichotomy is still popular in the latest study (Gao et al., 2020; Figure 2). Moreover, besides macrophages, there are other immune cells being discussed to play their respective promising roles in osteoimmunology-mediated osseointegration as well, such as T lymphocytes (Singhatanadgit et al., 2019) and mast cells (Zizzi et al., 2011; Marcatti Amarú Maximiano et al., 2017).

### Osteogenesis

Dental implant is most widely contacted with bone tissue. Osseointegration, the fundamental theory of modern oral implantology, is the process of establishing a direct connection between ordered bone tissue and surface of a loaded-implant without soft tissue involvement. Osseointegration starts with inserting implant into the drilled hole and obtains passive and mechanical primary stability between interfacial bone and biomaterial surface (Lee and Bance, 2019). The biological responses are activated afterward. As soon as the dental implant is implanted into bone tissue, the peri-implant area becomes congested and immediately some biomolecules from surrounding blood and interstitial fluid are absorbed to the implant surface to form a temporary bioactive layer for preparation of cell reaction, with bone marrow cells scattering around. MSCs and osteoblasts are stimulated and facilitated to migrate to osteogenic area on the surface of dental implants and start to proliferate and differentiate thus mineralization procedure is initiated. Simultaneously, osteoclasts are activated to drive bone resorption process after the formation of woven bone to replace it by lamellar bone with a higher degree of mineralization and load intensity (Lee and Bance, 2019). During the terminal stage of osteogenesis process, osteocytes and the surface of implant are directly contiguous with or without the dendritic structures of osteocytes, building a bioactive network in bone-implant area, which probably suggest the structural basis of osseoperception (Du et al., 2016). To harmonize the whole dynamic process of bone formation as well as bone resorption, bone remodeling cell populations including bone marrow mesenchymal stem cells (BMSCs), osteoblasts, osteoclasts and osteocytes etc. detect and translate biological signals in microenvironment then react to the given cues. The tight communication and multiple crosstalk among different cell populations are involved as well (Lotz et al., 2018; Shen et al., 2019; Sims and Martin, 2020; Tilkin et al., 2020; Wang H. et al., 2020). Therefore, it is of great significance to study the biochemistry and physiology phenomenon and, more importantly, the regulation mechanism on bone-implant interface to reach a clinical-friendly osseointegration.

### Angiogenesis

Bone tissue is well known as a high vascularization tissue. A normal vascular structure and a favorable microcirculation contribute to the health of peri-implant bone tissue and a clinical-friendly osseointegration, since the microvasculature transports nutrients and metabolites and provides a pivotal microenvironment for migration, proliferation and differentiation of osteogenesis-related cells. Saghiri et al. (2016) reviewed researches on effects of titanium alloys and surface characteristics and treatments of dental implants on angiogenesis process and highlighted that pro-angiogenic surface played a pivotal role in facilitating osseointegration.

A healthy and functional microvascular network attributes to promising vessel sprouting and vascular tube extension, respectively, under specification of tip cells and stalk cells, the two main populations of vascular endothelial cells (ECs), which are equally necessary in angiogenesis and vessel remodeling. Moreover, the process of angiogenesis involves several signals and pathways, mainly including growth factors (VEGF, FGF, PDGF, TGF-β, et al.), Notch signaling, MMPs and so on (Potente et al., 2011).

## Role of Hippo-YAP Signaling in Osteogenesis, Angiogenesis and Osteoimmunology

### Hippo-YAP Upstream Signals and Downstream Responses

Hippo-YAP responds to a variety of upstream signals in both intracellular and extracellular microenvironment. Hippo-YAP takes part in cell biomechanical respond mechanism to ECM, specifically, low ECM resistance leads to inhibiting activation of YAP/TAZ while high ECM resistance stimulates YAP/TAZ nuclear translocation (Totaro et al., 2017, 2018b; Moya and Halder, 2019). What’s more, some extracellular lipids or hormone signals can bond to G-protein-coupled receptors (GPCRs) and regulates Hippo-YAP signaling mediated by F-actin signaling (Moya and Halder, 2019). Based on a series of researches on the crosstalk between lysophosphatidic acid (LPA) and Hippo-YAP signaling, it is suggested that LPA is an upstream signal of Hippo pathway, binding to GPCRs and regulating the activity of the downstream effector YAP, which further mediates gene expression and cell behaviors (Yu et al., 2012; Moroishi et al., 2015; Park et al., 2016; Wang X. et al., 2018). In addition, Wnt signaling, cell polarity and metabolic property also enrich the transduction mechanism of Hippo-YAP signaling pathway (Totaro et al., 2018b; Xiang et al., 2018; Moya and Halder, 2019).

After dephosphorylation and nucleus translocation, YAP bonds on the enhancer of the target gene with TEAD1-4 to regulate gene transcription. It has been revealed by ChIP-seq data that AP-1 is also widely recruited to transcription regulatory sequences with YAP/TAZ-TEAD complex (Totaro et al., 2018b). By regulating expression of key genes, Hippo-YAP signaling pathway plays a versatile and significant regulatory role in organ development and regeneration in mammal, indicating their potential impacts on osseointegration induced by osteogenesis, angiogenesis and osteoimmunology.

### The Influence of Hippo-YAP Signaling on Osteogenesis

There is strong evidence indicates that BMSCs are regulated by some biological signals on differentiation into bone tissue via Hippo-YAP pathway. The knockout of GNAS activates Hippo signaling pathway and eventually suppresses osteogenic differentiation of BMSCs (An et al., 2019), consistently, through the regulation of Hippo-YAP signaling pathway RAMP1 can promote the osteogenesis process of BMSCs that induced by CGRP (Zhang et al., 2019). Tang and Weiss (2017) revealed an interaction of Snail/Slug and YAP/TAZ, which regulates the differentiation ability of BMSCs in a cooperative way, suggesting a potential impact on the properties of osteogenesis. In addition, it is lately identified that Hippo pathway plays an important role in the competing adipo-osteogenic differentiation of MSCs and it turns out that YAP promotes osteogenic differentiation and in contrast it antagonizes adipogenic differentiation (Lorthongpanich et al., 2019). However, Seo et al. (2013) reported that SOX2-YAP1 axis significantly contributed to maintained stemness and inhibited osteoblastic differentiation of BMSCs, which may be attributed to Dkk1-mediated repression of Wnt signaling induced by YAP1/β-catenin.

Hippo-YAP signaling pathway is also involved in osteoblast differentiation through multiple biological mechanisms. The suppression of YAP may induce lowering ligand bmp2a in MSCs, leading to a severe damage of Bmp signals in osteoblasts nearby, which downregulates osteoblast differentiation through a cell non-autonomous way (Brandão et al., 2019). In a recent vivo study it is demonstrated that suppression of OLFML1, a missense mutant gene in patients with congenital scoliosis, leads to the nuclear translocation of YAP thus promoting expression of target genes and results in an accelerated mineralization process of osteoblasts, suggesting OLFML1 inhibits bone development through a biomechanical mechanism dependent on Hippo-YAP signaling pathway in osteoblasts (Murakami et al., 2018). Despite a certain amount of evidence has been shown to support its pivotal functions in bone metabolism and osteogenesis process, the multiple effects of Hippo-YAP signaling pathway in osteoblast differentiation are still controversial and ambiguous and need to be further clarified. It is lately indicated that Ti ions toxicity impair surrounding bone tissue by inducing dephosphorylation of YAP and its expression in nuclear to suppress osteogenic differentiation of osteoblasts, likewise knockdown of YAP expression leads to rescuing the harm of toxic Ti ions from implants, though Hippo-YAP is definitely not the only underlying biological cue responded by osteoblasts (Zhu et al., 2018).

The dynamic balance between bone formation and resorption plays a pivotal role in a favorable osteogenesis and mounting evidence has indicated regulatory effects of Hippo-YAP on osteoclastogenesis and resorption activity to influence bone homeostasis. Deficiency of MST2 in bone marrow-derived macrophages (BMMs) exhibits increased osteoclast differentiation and conversely MST2 overexpression inhibits it (Lee et al., 2015). Zhao et al. confirm that inhibition of YAP1 and its association with the main transcriptional factor TEADs weakens formation and osteoclastic resorption of osteoclasts, as well as NF-κB signaling induced by RANKL, a mainly investigated signaling pathway that regulates osteoclast differentiation in previous study (Yang et al., 2018; Zhao et al., 2018). Moreover, Limd1 is considered as an important negative regulator of Hippo pathway (Thakur et al., 2010; Jagannathan et al., 2016). It has been concluded recently *in vitro* that *Polygonatum sibiricum* polysaccharide (PSP) suppresses Hippo-YAP pathway to inhibit differentiation of osteoclasts from BMMs through downregulating expression of miRNA-1224, of which the target gene is Limd1 (Li B. et al., 2019). Additionally, the further mechanism of Hippo-YAP signaling regulating osteoclastogenesis is probably implicated to the transcriptional regulation by TEAD1 that binds to an upstream enhancer element of osteoprotegerin (OPG) and promotes its expression, which was revealed in human periodontal ligament cells (PDLCs) (Li Q. et al., 2019). Although PDLCs are supposed to be the absent cell population in bone-implant interface tissue, however, there’s an increasing number of researches that uncover the significance of retaining PDL in the socket after tooth extraction and the promising contribution of PDLCs to implant osseointegration and bone repair (Pei et al., 2017; Washio et al., 2018; Karimi Dastgerdi et al., 2020). Osteoclastogenesis may also be partially dependent on potential YAP-GDF15 mechanism since in a recent study GDF-15 has been demonstrated to induce NF-κB activation in monocytic macrophages which contributes to further formation of osteoclasts and YAP has been indicated as an upstream signal of GDF15 in human PDLCs (Li et al., 2020).

Crosstalk between osteoblasts and osteoclasts can influence bone remodeling in a cell non-autonomous way. It is uncovered in a recent study that mechanical sensing protein PIEZO1 in osteoblastic cells promotes nuclear translocation of YAP and increases type II and IX collagens expression through PIEZO1-YAP axis mechanistically, causing inhibition of osteoclast resorption activity (Wang L. et al., 2020).

Hippo-YAP signaling in osteocytes seems to be poorly understood in relevant fields. While osteocytes interconnect and communicate with each other on the basis of lacunar/canalicular system, playing a pivotal role in bone metabolism and remodeling. Besides, during the terminal stage of osteogenesis process in osseointegration, osteocytes contribute to compensating the microenvironment of periodontal ligament around nature teeth, at least partially, providing a cushion for masticatory forces and inducing osseoperception (Du et al., 2016). Therefore, it is of great value to uncover the molecular mechanisms that control osteocytes-induced osseointegration thus improving the survival rate and long-term stability of implants. Kegelman et al. clarified that deficiency of YAP/TAZ in osteocytes impaired bone accrual, matrix collagen and mechanical intensity *in vivo*, which was mediated by perilacunar/canalicular weakened remodeling, indicating a perspective target for future study (Kegelman et al., 2020). However, the specific role that Hippo-YAP signaling plays and other involved signals and pathways remain to be further clarified.

### Hippo-YAP Impacts on Angiogenesis

#### Vessel Sprouting

YAP/TAZ promote sprouting angiogenesis by contributing to activity and function of vascular tip cells. Mechanistically, YAP/TAZ promotes migration of tip cells by activating CDC42 and facilitates the formation and junction of filopodia, an essential dynamic structure of tip cell that dominates its sprouting function, by promoting the remodeling of actin cytoskeleton (Kim et al., 2017; Sakabe et al., 2017). Likewise, the identity of tip cells are induced by CCN1, through interaction with integrin αvβ3/VEGFR and activation of downstream Hippo pathway thus promoting nucleus translocation of YAP/TAZ, which coordinates CCN1 in turn as a positive feedback (Park et al., 2019). While overactivating YAP/TAZ leads to pathologic sprouting pattern (Astone et al., 2018). Therefore, a proper regulatory effect of Hippo-YAP signaling plays a significant role in sprouting angiogenesis.

It’s also worth noticing the crosstalk between Hippo-YAP pathway and Notch signaling in regulating angiogenesis. Notch signaling plays a significant role in vessel sprouting (Pitulescu et al., 2017; Fournier et al., 2020). There are two interaction patterns that are mainly investigated between YAP/TAZ and Notch pathways: first, YAP/TAZ regulate Notch pathways by controlling gene expression level of Notch receptors and/or ligands through nuclear translocation of YAP/TAZ, inducing Notch signaling turning on in surrounding cells; second, YAP/TAZ and Notch intracellular domain (NICD) are co-activated to translocate to nuclear and co-regulate their common targets genes (Totaro et al., 2018a). Notch signaling has also been reported to interact with LPA, an upstream signaling of Hippo pathway, which is suggested to play a role in a series of cell functions including angiogenesis through Hippo-YAP signaling. Yasuda et al. figured out that endothelial LPA4 and LPA6, receptors that coupled with Gα12/Gα13, regulate expression of Notch ligand Dll4 via YAP/TAZ and play a crucial role in sprouting angiogenesis (Yasuda et al., 2019). In addition, Ren et al. (2019) identified that Notch1, one of the single-stranded transmembrane receptors of Notch pathway, may interact with LPA2 and mediate cell biological performances.

#### Extension of the Vascular Tube

The favorable proliferation capability of EC promotes extension of vascular tubes, mostly dependent on the proliferation of stalk cells (Potente et al., 2011). Hippo-YAP has also been discovered to regulate metabolism and proliferation activity in ECs.

As a major growth factor of vascular development, interaction mechanism of VEGF with Hippo-YAP pathway has been investigated in recent years. Mechanistically, the pro-angiogenic effect of VEGF is mediated by actin cytoskeleton activity, which triggers Hippo-YAP axis and transcriptional regulatory activity of YAP, targeting cell viability-related genes (Wang et al., 2017). Also, VEGF may activate Hippo-YAP pathway mediated by PI3K/MAPK signaling (Azad et al., 2018). Moreover, YAP/TAZ regulates metabolism and proliferation activity of ECs by promoting MYC signaling (Kim et al., 2017). However, Hippo-YAP shows opposing regulatory manner in hypoxic microenvironment. The viability and migration ability of ECs are promoted in myocardial infarction by miR-93, through suppressing LATS2 to inhibit Hippo-YAP pathway (Ma et al., 2020), which may be attributed to YAP/TAZ inactivating hypoxia-inducible factor 1α (HIF1α) signaling in ECs (Sivaraj et al., 2020). Therefore, more attention should be paid to the different regulatory roles of Hippo-YAP in different organs, especially the property in angiogenic osseointegration.

### Hippo-YAP Pathway Regulates Osteoimmunology

As major participants in osteoimmunology response, macrophages react to diverse biological signalings to adapt to different microenvironment, including Hippo-YAP signaling. Based on recent publications, it remains controversial on what kind of character YAP plays in macrophage polarization to M1/M2 phenotypes. It was reported that TGFβ1-mediated M2 polarization was facilitated by Wnt5a via stimulating YAP/TAZ (Feng et al., 2018). Consistently, Li C. et al. (2019) demonstrated *in vivo* that the expression profile of M1 phenotypic proinflammatory factors TNF-α and IL-1β were augmented while M2 characteristic anti-inflammatory factors IL-10 and TGF-β were weakened in myeloid-specific YAP knockout mice. Similar results can be found in tumor-associated macrophages (TAMs) as well (Huang et al., 2017; Jia et al., 2020; Zhao et al., 2020). However, some contrary results with regard to the regulatory effect of YAP in macrophages have been described. Zhou et al. pointed out that YAP promoted M1 but decreased M2 polarization based on the experimental results that specific knockout of myeloid YAP activated M2 polarization with IL-10 increase and IL-1β decrease but without any effect on production of TNF-α1, which is in conflict with the precious studies (Zhou et al., 2019). Additionally, Song et al. (2020) revealed the mechanism of YAP aggravating M1 phenotype in Kupffer cells that LPS-stimulated YAP upregulated expression of the classic proinflammatory cytokines including IL-6, TNF-α, and MCP-1 by binding to their promoter regions through association with its transcriptional factor TEADs.

Besides macrophages, there are other immune cells should be involved in this discussion, since they dominate early inflammatory response in the primary stage of osseointegration. While mostly relevant investigations rarely involve osseointegration procedure of foreign implants. Further study may uncover the role of Hippo-YAP in early peri-implant inflammatory response.

## Discussion and Future Outlook

### Potential Roles of LPA

LPA is a bioactive small ubiquitous lipid which naturally exists in the body and it contributes to a various of biological effects in nervous system, cardiovascular, cancer, immune system etc. (Choi et al., 2010; Yung et al., 2014). LPA’s unique physiological and pathological roles are revealed to be driven by extracellular signals through particular GPCRs which are called LPA1-6 (Choi et al., 2010). Specifically, the regulation effects of LPA on bone metabolism are mainly mediated by LPA1, LPA3, and LPA4 (Liu et al., 2010; Chen et al., 2019; Wu et al., 2019; Alioli et al., 2020); LPA4 and LPA6 play a facilitating role in developmental angiogenesis and LPA1 and LPA3 are found to mediate LPA/PKD-1-CD36 axis regulating proangiogenic and proarteriogenic reprogramming and *de novo* arteriogenesis (Ren et al., 2016; Dong et al., 2017; Yasuda et al., 2019); it is also worth noticing that LPA contributes to the formation of macrophages from monocytes in both mice and humans (Ray and Rai, 2017) and promotes LPA1 and LPA3 mediated conversion to foam cells (Chen et al., 2017). Numerous LPA-induced biological effects have been described and those cooperative and antagonistic signaling regulates cell activity in a highly complex manner. The investigations around LPA also suggest the potential role of LPA on osteogenesis, angiogenesis and osteoimmunology which may facilitate osseointegration procedure of implants, while its specific mechanism remains to be further clarified.

**FIGURE 3:**
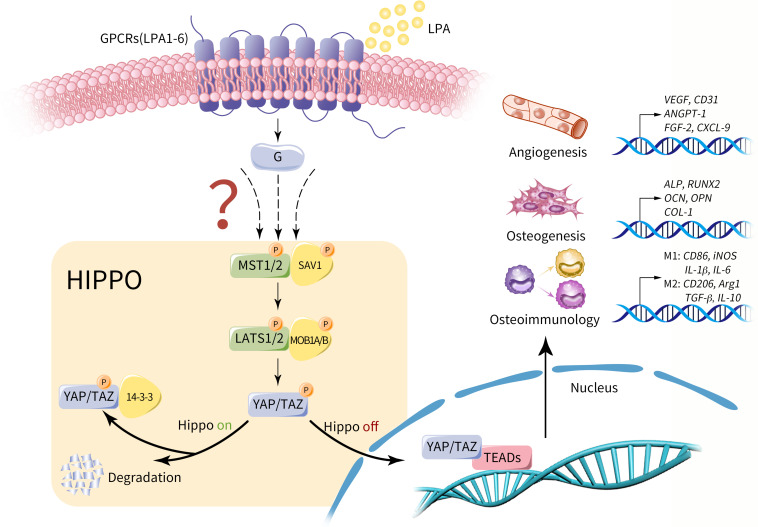
Potential role of LPA on Hippo-YAP mediated osteogenesis, angiogenesis and osteoimmunology. LPA may act as an upstream extracellular signal of Hippo pathway via GPCRs (LPA1-6). Provided that Hippo pathway is activated, MST1/2 and SAV1 are phosphorylated and activate the complex of LATS1/2 and MOB1A/B. As a result, the phosphorylated YAP/TAZ is sequestrated in the cytoplasm by interacting with 14-3-3 proteins or degraded. Conversely, in the context of Hippo off, YAP/TAZ is dephosphorylated and translocate into nucleus, regulating a variety of cell properties by influencing gene expression with its main transcriptional factor TEAD, including genes that dominate osteogenesis, angiogenesis and osteoimmunology. Therefore, we propose that LPA-Hippo axis may perform regulatory effects on osseointegration procedure by influencing osteogenesis, angiogenesis and osteoimmunology.

The previous study has showed the evidence on the downstream signaling pathway and cellular functions of LPA, whereby we further suppose that LPA may act as an upstream signal of Hippo pathway and promote LPA-Hippo axis mediated osteogenesis, angiogenesis and osteoimmunology, thus facilitating osseointegration process of implants or bone defect repair effectively (Figure 3). The potential mechanism around the series of molecular events remains controversial, which may suggest a prospective future research direction in involved fields.

### Friend or Foe? The Discrepancy Around Hippo-YAP Regulatory Effects on Osteogenesis-Related Cells

As mentioned above, the existing researches have revealed some inconsistent results on role of YAP that it represses osteogenic behaviors of MSCs and osteoblasts according to some reports. Friend or foe? The osteogenic potential of Hippo-YAP signaling still remains as a highly controversial and ambiguous issue. Here, we analyze the possible influencing factors that raise the discrepancy and suppose the potential regulatory pattern of Hippo-YAP in osteogenesis-related cells.

First of all, Hippo-YAP pathway may play an inconsistent role in osteogenic cells of different maturity stage and the osteogenic impact of YAP is maturity-dependent, which kind of explain the negative results in osteogenic cells of early maturity stages. Xiong et al. (2018) observed that in mesenchymal progenitors and osteoblasts of early stage YAP/TAZ suppressed their differentiation to mature osteoblasts and led to decreased bone mass, whereas, osteoblast popularity and bone formation were promoted by YAP/TAZ expression in mature osteoblasts and osteocytes. Seo et al. (2013) and Xiong et al. (2018) suggested that the negative osteogenic effects were attributed to the impaired Wnt signaling by YAP.

What’s more, the discrepancy around Hippo-YAP regulatory impacts may be caused by different properties of microenvironment. Yang et al. mimicked inflammatory microenvironment *in vitro* with TNF-α, the classic pro-inflammatory cytokine, then discovered an upregulated expression of pro-inflammatory IL-6 and RANKL and a reduced expression of anti-inflammatory OPG after knockdown of YAP1 in MC3T3-E1 cells (Yang et al., 2020). However, during the natural development procedure of mice, YAP knockdown in mature osteoblasts and osteocytes did not lead to changes on the expression of OPG or RANKL (Xiong et al., 2018). In a word, these discoveries suggest that the opposing regulatory role of YAP may due to the discrepancy between healthy and inflammatory local bone microenvironment, while further evidences should be shown to prove this inference.

In addition, Hippo-YAP is definitely not the only signaling pathway that plays pivotal roles in bone metabolism and regeneration (Ramasamy et al., 2016; Chen et al., 2018; Aslani et al., 2019; Luo et al., 2019; Maeda et al., 2019; Wang P. et al., 2019). In fact, osteogenesis is a complex and but orderly procedure induced and regulated by multiple synergistic and antagonistic effects.

Last but not least, it’s worth noticing that Hippo-YAP regulates cellular osteogenic function in not only cell autonomous mode but also non-autonomous mode (Brandão et al., 2019; Wang L. et al., 2020), which further contributes to the complexity of the working manner of Hippo-YAP pathway.

Taking all these factors into consideration, it’s hard to reach a consensus on the main reasons that cause the discrepancy around Hippo-YAP regulatory effect on osteogenesis-related cells. A compelling explanation will provide a deeper understanding of Hippo-YAP and indicate promising future research directions. We also suggest that researchers focus more on the conditions of Hippo-YAP promoting osteogenesis in future study, which is of great significance for further clarifying the multiple effects of Hippo-YAP pathway as well as exploring the potential clinical value.

### The Controversial Issues on Roles of YAP in Macrophages Polarization

YAP was proved to play controversial roles in macrophages polarization in mainstream studies as mentioned before and these inconsistent and ambiguous results raise our great interests in the dynamic regularity of macrophages YAP expression in inflammatory tissues as well as its deeper and further significances. Here, we analyze possible influencing factors and highlight potential future study directions.

Firstly, there is high heterogeneity among macrophages from distinct origins including M1 and M2 characteristics, indicating potential effects on the accuracy of experimental results. Macrophages widely exist in organs and tissues of the body, with high heterogeneity among specific subpopulations. Bone marrow derived macrophages (BMMs) are differentiated from monocytes with hematopoietic system origin, while as tissue−resident macrophages, peritoneal macrophages (PEMs) are indicated to be independent of hematopoietic progenitors and originated from yolk sac in recent works (Cain et al., 2013; Davies and Taylor, 2015). However, we notice that BMMs and PEMs were used asynchronously to conclude the deficiency of YAP in macrophages induced macrophage polarization to M2 while the activation of M1 macrophages was suppressed, whereby suggesting therapeutic impact on inflammatory microenvironment (Zhou et al., 2019). In fact, the sources macrophages derived from may have an impact on polarizing signatures, since the different expression levels of M1/M2-related genes in BMMs and PEMs have been proved, including both surface markers and soluble mediators, which, respectively, indicate discrepancies in phenotype and cellular function (Bisgaard et al., 2016). Besides, properties of aging-related phagocytosis and immigration in 3D microenvironment were compared between BMMs and PEMs and the two subpopulations presented inconsistent results (Cougoule et al., 2012; Linehan et al., 2014). Therefore, it is of value to reevaluate the inherent differences between distinct original macrophages to promote convincingness and applicability of involved researches, especially the ones with regard to polarization tendency into M1 or M2. Nevertheless, the proper cell model to simulate macrophage polarization and figure out its role in implicated diseases still needs to be further clarified.

Additionally, the classic M1/M2 dichotomy has been enriched in study on macrophage. As major participants in inflammatory response, macrophages show diverse phenotypic and functional differences reacting to different microenvironment. Based on the stimuli, macrophages are divided into classically activated macrophages (M1) and alternatively activated macrophages (M2) according to the existing classification model, with specific cytokine profile and biologic functions, respectively. In fact, as two extreme activated states, M1 and M2 rarely exist in microenvironment since macrophages are actually polarizing along the polarization spectrum and present some transitional state signatures with both M1 and M2 hallmarks (Davies and Taylor, 2015; Brown et al., 2017). In recent years, based on M1/M2 dichotomy, researchers have identified more subtypes of macrophages and expanded the definition of macrophage category, since as two extremes of a continuum and there are strong biochemistry and physiology differences between M1 and M2. For instance, M2 macrophages are further categorized into M2a, M2b, and M2c, the subtypes, respectively, activated by IL-4 and IL-13, immune complexes and LPS, and IL-10, presenting different biologic characteristics and effects, respectively (Mantovani et al., 2004). Additionally, there are more classifying patterns to describe a specific macrophage population that have been proposed to obtain more precise descriptions, such as CD169^+^ and TCR^+^ macrophages (Chávez-Galán et al., 2015). In conclusion, the previous studies have showed an enrichment of the classic M1/M2 dichotomy and choosing a suitable classifying pattern can be taken in to consideration in future researches.

## Conclusion

In this review we suggest that a clinical-friendly osseointegration is essentially determined by favorable osteogenesis, angiogenesis and osteoimmunology, with a complex series of peri-implant cellular and molecular events happening during those procedures. Moreover, Hippo-YAP signaling pathway plays pivotal multiple regulatory roles in osteogenesis, angiogenesis and osteoimmunology. In short, the potential effects of Hippo-YAP on promoting osseointegration will contribute to the discovery of prospective clinical therapy.

## Author Contributions

AZ and HY designed the outline and drafted and critically revised the manuscript. JL designed the figures. JZ, YJ, and BW contributed to drafting the manuscript. LX contributed to designing and critically revising the manuscript. All authors contributed to the article and approved the submitted version.

## Conflict of Interest

The authors declare that the research was conducted in the absence of any commercial or financial relationships that could be construed as a potential conflict of interest.

## References

[B1] Alioli C. A. Demesmay L. Laurencin-Dalacieux S. Beton N. Farlay D. Follet H. (2020). Expression of the type 1 lysophosphatidic acid receptor in osteoblastic cell lineage controls both bone mineralization and osteocyte specification. *Biochim. Biophys. Acta* 1865:158715. 10.1016/j.bbalip.2020.158715 32330664

[B2] An J. Li G. Zhang J. Zhou H. Jiang J. Wang X. (2019). GNAS knockdown suppresses osteogenic differentiation of mesenchymal stem cells via activation of Hippo signaling pathway. *J. Cell. Physiol.* 234 22299–22310. 10.1002/jcp.28796 31148202

[B3] Arron J. R. Choi Y. (2000). Bone versus immune system. *Nature* 408 535–536. 10.1038/35046196 11117729

[B4] Aslani S. Abhari A. Sakhinia E. Sanajou D. Rajabi H. Rahimzadeh S. (2019). Interplay between microRNAs and Wnt, transforming growth factor-β, and bone morphogenic protein signaling pathways promote osteoblastic differentiation of mesenchymal stem cells. *J. Cell. Physiol.* 234 8082–8093. 10.1002/jcp.27582 30548580

[B5] Astone M. Lai J. K. H. Dupont S. Stainier D. Y. R. Argenton F. Vettori A. (2018). Zebrafish mutants and TEAD reporters reveal essential functions for Yap and Taz in posterior cardinal vein development. *Sci. Rep.* 8:10189. 10.1038/s41598-018-27657-x 29976931 PMC6033906

[B6] Azad T. Janse van Rensburg H. J. Lightbody E. D. Neveu B. Champagne A. Ghaffari A. (2018). A LATS biosensor screen identifies VEGFR as a regulator of the Hippo pathway in angiogenesis. *Nat. Commun.* 9:1061. 10.1038/s41467-018-03278-w 29535383 PMC5849716

[B7] Bai L. Du Z. Du J. Yao W. Zhang J. Weng Z. (2018a). A multifaceted coating on titanium dictates osteoimmunomodulation and osteo/angio-genesis towards ameliorative osseointegration. *Biomaterials* 162 154–169. 10.1016/j.biomaterials.2018.02.010 29454274

[B8] Bai L. Liu Y. Du Z. Weng Z. Yao W. Zhang X. (2018b). Differential effect of hydroxyapatite nano-particle versus nano-rod decorated titanium micro-surface on osseointegration. *Acta Biomater.* 76 344–358. 10.1016/j.actbio.2018.06.023 29908975

[B9] Barboza-Solís C. Porras-Chaverri M. Fantin R. (2019). Is tooth loss important when evaluating perceived general health? Findings from a nationally representative study of Costa Rican adults. *Community Dent. Oral Epidemiol.* 47 358–365. 10.1111/cdoe.12466 31074536

[B10] Bisgaard L. S. Mogensen C. K. Rosendahl A. Cucak H. Nielsen L. B. Rasmussen S. E. (2016). Bone marrow-derived and peritoneal macrophages have different inflammatory response to oxLDL and M1/M2 marker expression – implications for atherosclerosis research. *Sci. Rep.* 6:35234. 10.1038/srep35234 27734926 PMC5062347

[B11] Bollman M. Malbrue R. Li C. Yao H. Guo S. Yao S. (2020). Improvement of osseointegration by recruiting stem cells to titanium implants fabricated with 3D printing. *Ann. N. Y. Acad. Sci.* 1463 37–44. 10.1111/nyas.14251 31603258

[B12] Brandão A. S. Bensimon-Brito A. Lourenço R. Borbinha J. Soares A. R. Mateus R. (2019). Yap induces osteoblast differentiation by modulating Bmp signalling during zebrafish caudal fin regeneration. *J. Cell Sci.* 132:jcs231993. 10.1242/jcs.231993 31636113

[B13] Brown B. N. Haschak M. J. Lopresti S. T. Stahl E. C. (2017). Effects of age-related shifts in cellular function and local microenvironment upon the innate immune response to implants. *Semin. Immunol.* 29 24–32. 10.1016/j.smim.2017.05.001 28539184 PMC5612855

[B14] Brunetti G. D’Amelio P. Mori G. Faienza M. F. (2020). Editorial: updates on osteoimmunology: what’s new on the crosstalk between bone and immune cells. *Front. Endocrinol.* 11:74. 10.3389/fendo.2020.00074 32153510 PMC7045046

[B15] Cain D. W. O’Koren E. G. Kan M. J. Womble M. Sempowski G. D. Hopper K. (2013). Identification of a tissue-specific, C/EBPβ-dependent pathway of differentiation for murine peritoneal macrophages. *J. Immunol.* 191 4665–4675. 10.4049/jimmunol.1300581 24078688 PMC3808250

[B16] Chávez-Galán L. Olleros M. L. Vesin D. Garcia I. (2015). Much More than M1 and M2 Macrophages, There are also CD169+ and TCR+ Macrophages. *Front. Immunol.* 6:263. 10.3389/fimmu.2015.00263 26074923 PMC4443739

[B17] Chen L. Zhang J. Deng X. Liu Y. Yang X. Wu Q. (2017). Lysophosphatidic acid directly induces macrophage-derived foam cell formation by blocking the expression of SRBI. *Biochem. Biophys. Res. Commun.* 491 587–594. 10.1016/j.bbrc.2017.07.159 28765047

[B18] Chen S. Guo Y. Liu R. Wu S. Fang J. Huang B. (2018). Tuning surface properties of bone biomaterials to manipulate osteoblastic cell adhesion and the signaling pathways for the enhancement of early osseointegration. *Colloids Surf. B Biointerfaces* 164 58–69. 10.1016/j.colsurfb.2018.01.022 29413621

[B19] Chen X. Song Z. Chen R. Tan S. Huang C. Liu Y. (2019). Lysophosphatidic acid enhanced the osteogenic and angiogenic capability of osteoblasts via LPA1/3 receptor. *Connect. Tissue Res.* 60 85–94. 10.1080/03008207.2018.1439485 29447019

[B20] Choi J. W. Herr D. R. Noguchi K. Yung Y. C. Lee C.-W. Mutoh T. (2010). LPA receptors: subtypes and biological actions. *Annu. Rev. Pharmacol. Toxicol.* 50 157–186. 10.1146/annurev.pharmtox.010909.105753 20055701

[B21] Cooper L. Tarnow D. Froum S. Moriarty J. De Kok I. (2016). Comparison of marginal bone changes with internal conus and external hexagon design implant systems: a prospective, randomized study. *Int. J. Periodontics Restorative Dent.* 36 631–642. 10.11607/prd.2433 27560668

[B22] Cougoule C. Van Goethem E. Le Cabec V. Lafouresse F. Dupré L. Mehraj V. (2012). Blood leukocytes and macrophages of various phenotypes have distinct abilities to form podosomes and to migrate in 3D environments. *Eur. J. Cell Biol.* 91 938–949. 10.1016/j.ejcb.2012.07.002 22999511

[B23] Davies L. C. Taylor P. R. (2015). Tissue-resident macrophages: then and now. *Immunology* 144 541–548. 10.1111/imm.12451 25684236 PMC4368161

[B24] Dohle E. Bischoff I. Böse T. Marsano A. Banfi A. Unger R. E. (2014). Macrophage-mediated angiogenic activation of outgrowth endothelial cells in co-culture with primary osteoblasts. *Eur. Cell. Mater.* 27 149–165. 10.22203/eCM.v027a12 24554272

[B25] Dong L. Yuan Y. Opansky C. Chen Y. Aguilera-Barrantes I. Wu S. (2017). Diet-induced obesity links to ER positive breast cancer progression via LPA/PKD-1-CD36 signaling-mediated microvascular remodeling. *Oncotarget* 8 22550–22562. 10.18632/oncotarget.15123 28186980 PMC5410244

[B26] Du Z. Ivanovski S. Hamlet S. M. Feng J. Q. Xiao Y. (2016). The ultrastructural relationship between osteocytes and dental implants following osseointegration: osteocytes and dental implants. *Clin. Implant Dent. Relat. Res.* 18 270–280. 10.1111/cid.12257 25039329

[B27] Feng Y. Liang Y. Zhu X. Wang M. Gui Y. Lu Q. (2018). The signaling protein Wnt5a promotes TGFβ1-mediated macrophage polarization and kidney fibrosis by inducing the transcriptional regulators Yap/Taz. *J. Biol. Chem.* 293 19290–19302. 10.1074/jbc.RA118.005457 30333225 PMC6302175

[B28] Fournier P. Viallard C. Dejda A. Sapieha P. Larrivée B. Royal I. (2020). The protein tyrosine phosphatase PTPRJ/DEP-1 contributes to the regulation of the Notch-signaling pathway and sprouting angiogenesis. *Angiogenesis* 23 145–157. 10.1007/s10456-019-09683-z 31598898

[B29] Fukuda N. Tsuchiya A. Sunarso, Toita R. Tsuru K. Mori Y. (2019). Surface plasma treatment and phosphorylation enhance the biological performance of poly(ether ether ketone). *Colloids Surf. B Biointerfaces* 173 36–42. 10.1016/j.colsurfb.2018.09.032 30266018

[B30] Gao A. Liao Q. Xie L. Wang G. Zhang W. Wu Y. (2020). Tuning the surface immunomodulatory functions of polyetheretherketone for enhanced osseointegration. *Biomaterials* 230:119642. 10.1016/j.biomaterials.2019.119642 31787332

[B31] Gibon E. Lu L. Y. Nathan K. Goodman S. B. (2017). Inflammation, ageing, and bone regeneration. *J. Orthop. Transl.* 10 28–35. 10.1016/j.jot.2017.04.002 29094003 PMC5662134

[B32] Guder C. Gravius S. Burger C. Wirtz D. C. Schildberg F. A. (2020). Osteoimmunology: a current update of the interplay between bone and the immune system. *Front. Immunol.* 11:58. 10.3389/fimmu.2020.00058 32082321 PMC7004969

[B33] Hamlet S. Alfarsi M. George R. Ivanovski S. (2012). The effect of hydrophilic titanium surface modification on macrophage inflammatory cytokine gene expression. *Clin. Oral Implants Res.* 23 584–590. 10.1111/j.1600-0501.2011.02325.x 22093029

[B34] Hamlet S. Ivanovski S. (2011). Inflammatory cytokine response to titanium chemical composition and nanoscale calcium phosphate surface modification. *Acta Biomater.* 7 2345–2353. 10.1016/j.actbio.2011.01.032 21281745

[B35] Hankenson K. D. Dishowitz M. Gray C. Schenker M. (2011). Angiogenesis in bone regeneration. *Injury* 42 556–561. 10.1016/j.injury.2011.03.035 21489534 PMC3105195

[B36] Hickin M. P. Shariff J. A. Jennette P. J. Finkelstein J. Papapanou P. N. (2017). Incidence and determinants of dental implant failure: a review of electronic health records in a U.S. dental school. *J. Dent. Educ.* 81 1233–1242. 10.21815/jde.017.080 28966189

[B37] Howe M.-S. Keys W. Richards D. (2019). Long-term (10-year) dental implant survival: a systematic review and sensitivity meta-analysis. *J. Dent.* 84 9–21. 10.1016/j.jdent.2019.03.008 30904559

[B38] Huang Y.-J. Yang C.-K. Wei P.-L. Huynh T.-T. Whang-Peng J. Meng T.-C. (2017). Ovatodiolide suppresses colon tumorigenesis and prevents polarization of M2 tumor-associated macrophages through YAP oncogenic pathways. *J. Hematol. Oncol.* 10:60. 10.1186/s13045-017-0421-3 PMC5329923 28241877

[B39] Insua A. Monje A. Wang H.-L. Miron R. J. (2017). Basis of bone metabolism around dental implants during osseointegration and peri-implant bone loss. *J. Biomed. Mater. Res. A* 105 2075–2089. 10.1002/jbm.a.36060 28281321

[B40] Jagannathan R. Schimizzi G. V. Zhang K. Loza A. J. Yabuta N. Nojima H. (2016). AJUBA LIM proteins limit Hippo activity in proliferating cells by sequestering the Hippo core kinase complex in the cytosol. *Mol. Cell Biol.* 36 2526–2542. 10.1128/mcb.00136-16 27457617 PMC5038147

[B41] Jia J. Zhang H. He L. Zhang H. Shu M. (2020). Cutaneous neurofibroma cells with active YAP promotes proliferation of macrophages resulting in increased accumulation of macrophages by modulating CCL5 and TGF-β1. *Oncol. Rep.* 43 1319–1330. 10.3892/or.2020.7513 32323813

[B42] Karimi Dastgerdi A. Rouhi G. Dehghan M. M. Farzad-Mohajeri S. Barikani H. R. (2020). Linear momenta transferred to the dental implant-bone and natural tooth—PDL-bone constructs under impact loading: a comparative *in-vitro* and *in-silico* Study. *Front. Bioeng. Biotechnol.* 8:544. 10.3389/fbioe.2020.00544 32596223 PMC7303479

[B43] Kegelman C. D. Coulombe J. C. Jordan K. M. Horan D. J. Qin L. Robling A. G. (2020). YAP and TAZ mediate osteocyte perilacunar/canalicular remodeling. *J. Bone Miner. Res.* 35 196–210. 10.1002/jbmr.3876 31610061 PMC7066596

[B44] Kim J. Kim Y. H. Kim J. Park D. Y. Bae H. Lee D.-H. (2017). YAP/TAZ regulates sprouting angiogenesis and vascular barrier maturation. *J. Clin. Invest.* 127 3441–3461. 10.1172/JCI93825 28805663 PMC5669570

[B45] Lee J. Youn B. U. Kim K. Kim J. H. Lee D. Seong S. (2015). Mst2 controls bone homeostasis by regulating osteoclast and osteoblast differentiation. *J. Bone Miner. Res.* 30 1597–1607. 10.1002/jbmr.2503 25761670

[B46] Lee J. W. Y. Bance M. L. (2019). Physiology of osseointegration. *Otolaryngol. Clin. North Am.* 52 231–242. 10.1016/j.otc.2018.11.004 30612758

[B47] Li B. Wu P. Fu W. Xiong Y. Zhang L. Gao Y. (2019). The role and mechanism of miRNA-1224 in the *Polygonatum sibiricum* polysaccharide regulation of bone marrow-derived macrophages to osteoclast differentiation. *Rejuvenation Res.* 22 420–430. 10.1089/rej.2018.2126 30632458

[B48] Li C. Jin Y. Wei S. Sun Y. Jiang L. Zhu Q. (2019). Hippo signaling controls NLR family pyrin domain containing 3 activation and governs immunoregulation of mesenchymal stem cells in mouse liver injury. *Hepatology* 70 1714–1731. 10.1002/hep.30700 31063235 PMC6819196

[B49] Li Q. Han G. Liu D. Zhou Y. (2019). Force-induced decline of TEA domain family member 1 contributes to osteoclastogenesis via regulation of Osteoprotegerin. *Arch. Oral Biol.* 100 23–32. 10.1016/j.archoralbio.2019.01.020 30771694

[B50] Li S. Li Q. Zhu Y. Hu W. (2020). GDF15 induced by compressive force contributes to osteoclast differentiation in human periodontal ligament cells. *Exp. Cell Res.* 387:111745. 10.1016/j.yexcr.2019.111745 31765611

[B51] Linehan E. Dombrowski Y. Snoddy R. Fallon P. G. Kissenpfennig A. Fitzgerald D. C. (2014). Aging impairs peritoneal but not bone marrow-derived macrophage phagocytosis. *Aging Cell* 13 699–708. 10.1111/acel.12223 24813244 PMC4326936

[B52] Liu Y.-B. Kharode Y. Bodine P. V. N. Yaworsky P. J. Robinson J. A. Billiard J. (2010). LPA induces osteoblast differentiation through interplay of two receptors: LPA1 and LPA4. *J. Cell. Biochem.* 109 794–800. 10.1002/jcb.22471 20069565

[B53] Lorthongpanich C. Thumanu K. Tangkiettrakul K. Jiamvoraphong N. Laowtammathron C. Damkham N. (2019). YAP as a key regulator of adipo-osteogenic differentiation in human MSCs. *Stem Cell Res. Ther.* 10:402. 10.1186/s13287-019-1494-4 31852542 PMC6921580

[B54] Lotz E. M. Berger M. B. Schwartz Z. Boyan B. D. (2018). Regulation of osteoclasts by osteoblast lineage cells depends on titanium implant surface properties. *Acta Biomater.* 68 296–307. 10.1016/j.actbio.2017.12.039 29292169 PMC5803380

[B55] Luo Z. Shang X. Zhang H. Wang G. Massey P. A. Barton S. R. (2019). Notch signaling in osteogenesis, osteoclastogenesis, and angiogenesis. *Am. J. Pathol.* 189 1495–1500. 10.1016/j.ajpath.2019.05.005 31345466 PMC6699068

[B56] Ma C. Peng P. Zhou Y. Liu T. Wang L. Lu C. (2020). MicroRNA-93 promotes angiogenesis and attenuates remodeling via inactivation of the Hippo/Yap pathway by targeting Lats2 after myocardial infarctionω. *Mol. Med. Rep.* 22 483–493. 10.3892/mmr.2020.11085 32319642 PMC7248469

[B57] Ma Q. Fang L. Jiang N. Zhang L. Wang Y. Zhang Y. (2018). Bone mesenchymal stem cell secretion of sRANKL/OPG/M-CSF in response to macrophage-mediated inflammatory response influences osteogenesis on nanostructured Ti surfaces. *Biomaterials* 154 234–247. 10.1016/j.biomaterials.2017.11.003 29144982

[B58] Maeda K. Kobayashi Y. Koide M. Uehara S. Okamoto M. Ishihara A. (2019). The regulation of bone metabolism and disorders by Wnt signaling. *Int. J. Mol. Sci.* 20:5525. 10.3390/ijms20225525 31698687 PMC6888566

[B59] Mantovani A. Sica A. Sozzani S. Allavena P. Vecchi A. Locati M. (2004). The chemokine system in diverse forms of macrophage activation and polarization. *Trends Immunol.* 25 677–686. 10.1016/j.it.2004.09.015 15530839

[B60] Marcatti Amarú Maximiano W. Marino Mazucato V. Tambasco de Oliveira P. Célia Jamur M. Oliver C. (2017). Nanotextured titanium surfaces stimulate spreading, migration, and growth of rat mast cells: nanotextured titanium surfaces stimulate mast cells. *J. Biomed. Mater. Res. A* 105 2150–2161. 10.1002/jbm.a.36076 28371254

[B61] Moroishi T. Park H. W. Qin B. Chen Q. Meng Z. Plouffe S. W. (2015). A YAP/TAZ-induced feedback mechanism regulates Hippo pathway homeostasis. *Genes Dev.* 29 1271–1284. 10.1101/gad.262816.115 26109050 PMC4495398

[B62] Moya I. M. Halder G. (2019). Hippo-YAP/TAZ signalling in organ regeneration and regenerative medicine. *Nat. Rev. Mol. Cell Biol.* 20 211–226. 10.1038/s41580-018-0086-y 30546055

[B63] Murakami K. Kikugawa S. Kobayashi Y. Uehara S. Suzuki T. Kato H. (2018). Olfactomedin-like protein OLFML1 inhibits Hippo signaling and mineralization in osteoblasts. *Biochem. Biophys. Res. Commun.* 505 419–425. 10.1016/j.bbrc.2018.09.112 30266405

[B64] Okamoto K. Nakashima T. Shinohara M. Negishi-Koga T. Komatsu N. Terashima A. (2017). Osteoimmunology: the conceptual framework unifying the immune and skeletal systems. *Physiol. Rev.* 97 1295–1349. 10.1152/physrev.00036.2016 28814613

[B65] Palmquist A. Johansson A. Suska F. Brånemark R. Thomsen P. (2013). Acute inflammatory response to laser-induced micro- and nano-sized titanium surface features: inflammatory response to laser-modified titanium. *Clin. Implant Dent. Relat. Res.* 15 96–104. 10.1111/j.1708-8208.2011.00361.x 21745322

[B66] Park M.-H. Kim A. K. Manandhar S. Oh S.-Y. Jang G.-H. Li K. (2019). CCN1 interlinks integrin and hippo pathway to autoregulate tip cell activity. *eLife* 8:e46012. 10.7554/eLife.46012 31429823 PMC6726423

[B67] Park R. Moon U. Y. Park J. Y. Hughes L. J. Johnson R. L. Cho S.-H. (2016). Yap is required for ependymal integrity and is suppressed in LPA-induced hydrocephalus. *Nat. Commun.* 7:10329. 10.1038/ncomms10329 26754915 PMC4729961

[B68] Pei X. Wang L. Chen C. Yuan X. Wan Q. Helms J. A. (2017). Contribution of the PDL to osteotomy repair and implant osseointegration. *J. Dent. Res.* 96 909–916. 10.1177/0022034517707513 28481696 PMC5502960

[B69] Pitulescu M. E. Schmidt I. Giaimo B. D. Antoine T. Berkenfeld F. Ferrante F. (2017). Dll4 and Notch signalling couples sprouting angiogenesis and artery formation. *Nat. Cell Biol.* 19 915–927. 10.1038/ncb3555 28714968

[B70] Potente M. Gerhardt H. Carmeliet P. (2011). Basic and therapeutic aspects of angiogenesis. *Cell* 146 873–887. 10.1016/j.cell.2011.08.039 21925313

[B71] Ramasamy S. K. Kusumbe A. P. Itkin T. Gur-Cohen S. Lapidot T. Adams R. H. (2016). Regulation of hematopoiesis and osteogenesis by blood vessel–derived signals. *Annu. Rev. Cell Dev. Biol.* 32 649–675. 10.1146/annurev-cellbio-111315-124936 27576121

[B72] Ray R. Rai V. (2017). Lysophosphatidic acid converts monocytes into macrophages in both mice and humans. *Blood* 129 1177–1183. 10.1182/blood-2016-10-743757 28069607

[B73] Ren B. Best B. Ramakrishnan D. P. Walcott B. P. Storz P. Silverstein R. L. (2016). LPA/PKD-1-FoxO1 signaling axis mediates endothelial cell CD36 transcriptional repression and proangiogenic and proarteriogenic reprogramming. *Arterioscler. Thromb. Vasc. Biol.* 36 1197–1208. 10.1161/ATVBAHA.116.307421 27013613 PMC4882231

[B74] Ren Z. Zhang C. Ma L. Zhang X. Shi S. Tang D. (2019). Lysophosphatidic acid induces the migration and invasion of SGC-7901 gastric cancer cells through the LPA2 and Notch signaling pathways. *Int. J. Mol. Med.* 44 67–78. 10.3892/ijmm.2019.4186 31115486 PMC6559315

[B75] Saghiri M.-A. Asatourian A. Garcia-Godoy F. Sheibani N. (2016). The role of angiogenesis in implant dentistry part I: review of titanium alloys, surface characteristics and treatments. *Med. Oral Patol. Oral Cirugia Bucal* 21 e514–e525. 10.4317/medoral.21199 PMC4920467 27031073

[B76] Sakabe M. Fan J. Odaka Y. Liu N. Hassan A. Duan X. (2017). YAP/TAZ-CDC42 signaling regulates vascular tip cell migration. *Proc. Natl. Acad. Sci. U.S.A.* 114 10918–10923. 10.1073/pnas.1704030114 28973878 PMC5642684

[B77] Seo E. Basu-Roy U. Gunaratne P. H. Coarfa C. Lim D.-S. Basilico C. (2013). SOX2 regulates YAP1 to maintain stemness and determine cell fate in the osteo-adipo lineage. *Cell Rep.* 3 2075–2087. 10.1016/j.celrep.2013.05.029 23791527 PMC5053763

[B78] Shemtov-Yona K. Rittel D. (2015). An overview of the mechanical integrity of dental implants. *Biomed Res. Int.* 2015:547384. 10.1155/2015/547384 26583117 PMC4637045

[B79] Shen X. Yu Y. Ma P. Luo Z. Hu Y. Li M. (2019). Titania nanotubes promote osteogenesis via mediating crosstalk between macrophages and MSCs under oxidative stress. *Colloids Surf. B Biointerfaces* 180 39–48. 10.1016/j.colsurfb.2019.04.033 31028963

[B80] Shi M. Chen Z. Farnaghi S. Friis T. Mao X. Xiao Y. (2016). Copper-doped mesoporous silica nanospheres, a promising immunomodulatory agent for inducing osteogenesis. *Acta Biomater.* 30 334–344. 10.1016/j.actbio.2015.11.033 26596565

[B81] Shi M. Xia L. Chen Z. Lv F. Zhu H. Wei F. (2017). Europium-doped mesoporous silica nanosphere as an immune-modulating osteogenesis/angiogenesis agent. *Biomaterials* 144 176–187. 10.1016/j.biomaterials.2017.08.027 28837959

[B82] Sims N. A. Martin T. J. (2020). Osteoclasts provide coupling signals to osteoblast lineage cells through multiple mechanisms. *Annu. Rev. Physiol.* 82 507–529. 10.1146/annurev-physiol-021119-034425 31553686

[B83] Singhatanadgit W. Toso M. Pratheepsawangwong B. Pimpin A. Srituravanich W. (2019). Titanium dioxide nanotubes of defined diameter enhance mesenchymal stem cell proliferation via JNK- and ERK-dependent up-regulation of fibroblast growth factor-2 by T lymphocytes. *J. Biomater. Appl.* 33 997–1010. 10.1177/0885328218816565 30757966

[B84] Sivaraj K. K. Dharmalingam B. Mohanakrishnan V. Jeong H.-W. Kato K. Schröder S. (2020). YAP1 and TAZ negatively control bone angiogenesis by limiting hypoxia-inducible factor signaling in endothelial cells. *eLife* 9:e50770. 10.7554/eLife.50770 31958058 PMC6970532

[B85] Song K. Kwon H. Han C. Chen W. Zhang J. Ma W. (2020). Yes-associated protein in Kupffer cells enhances the production of pro-inflammatory cytokines and promotes the development of non-alcoholic steatohepatitis. *Hepatology* 72 72–87. 10.1002/hep.30990 31610032 PMC7153981

[B86] Tang Y. Weiss S. J. (2017). Snail/Slug-YAP/TAZ complexes cooperatively regulate mesenchymal stem cell function and bone formation. *Cell Cycle* 16 399–405. 10.1080/15384101.2017.1280643 28112996 PMC5351930

[B87] Thakur M. D. Feng Y. Jagannathan R. Seppa M. J. Skeath J. B. Longmore G. D. (2010). Ajuba LIM proteins are negative regulators of the Hippo signaling pathway. *Curr. Biol.* 20 657–662. 10.1016/j.cub.2010.02.035 20303269 PMC2855397

[B88] Tilkin R. G. Régibeau N. Lambert S. D. Grandfils C. (2020). Correlation between surface properties of polystyrene and polylactide materials and fibroblast and osteoblast cell line behavior: a critical overview of the literature. *Biomacromolecules* 21 1995–2013. 10.1021/acs.biomac.0c00214 32181654

[B89] Totaro A. Castellan M. Battilana G. Zanconato F. Azzolin L. Giulitti S. (2017). YAP/TAZ link cell mechanics to Notch signalling to control epidermal stem cell fate. *Nat. Commun.* 8:15206. 10.1038/ncomms15206 28513598 PMC5442321

[B90] Totaro A. Castellan M. Di Biagio D. Piccolo S. (2018a). Crosstalk between YAP/TAZ and Notch signaling. *Trends Cell Biol.* 28 560–573. 10.1016/j.tcb.2018.03.001 29665979 PMC6992418

[B91] Totaro A. Panciera T. Piccolo S. (2018b). YAP/TAZ upstream signals and downstream responses. *Nat. Cell Biol.* 20 888–899. 10.1038/s41556-018-0142-z 30050119 PMC6186418

[B92] Trindade R. Albrektsson T. Galli S. Prgomet Z. Tengvall P. Wennerberg A. (2018). Osseointegration and foreign body reaction: titanium implants activate the immune system and suppress bone resorption during the first 4 weeks after implantation. *Clin. Implant Dent. Relat. Res.* 20 82–91. 10.1111/cid.12578 29283206

[B93] Tsukasaki M. Takayanagi H. (2019). Osteoimmunology: evolving concepts in bone-immune interactions in health and disease. *Nat. Rev. Immunol.* 19 626–642. 10.1038/s41577-019-0178-8 31186549

[B94] Walsh M. C. Takegahara N. Kim H. Choi Y. (2018). Updating osteoimmunology: regulation of bone cells by innate and adaptive immunity. *Nat. Rev. Rheumatol.* 14 146–156. 10.1038/nrrheum.2017.213 29323344 PMC5821527

[B95] Wang H. Yang G. Xiao Y. Luo G. Li G. Li Z. (2020). Friend or foe? Essential roles of osteoclast in maintaining skeletal health. *Biomed Res. Int.* 2020:4791786. 10.1155/2020/4791786 32190665 PMC7073503

[B96] Wang J. Meng F. Song W. Jin J. Ma Q. Fei D. (2018). Nanostructured titanium regulates osseointegration via influencing macrophage polarization in the osteogenic environment. *Int. J. Nanomed.* 13 4029–4043. 10.2147/IJN.S163956 30022825 PMC6045901

[B97] Wang L. You X. Lotinun S. Zhang L. Wu N. Zou W. (2020). Mechanical sensing protein PIEZO1 regulates bone homeostasis via osteoblast-osteoclast crosstalk. *Nat. Commun.* 11:282. 10.1038/s41467-019-14146-6 31941964 PMC6962448

[B98] Wang P. Perche F. Logeart-Avramoglou D. Pichon C. (2019). RNA-based therapy for osteogenesis. *Int. J. Pharm.* 569:118594. 10.1016/j.ijpharm.2019.118594 31394184

[B99] Wang X. Hou H. Song K. Zhang Z. Zhang S. Cao Y. (2018). Lpar2b controls lateral line tissue size by regulating Yap1 activity in zebrafish. *Front. Mol. Neurosci.* 11:34. 10.3389/fnmol.2018.00034 29479307 PMC5812253

[B100] Wang Y. Zhang Y. Sculean A. Bosshardt D. D. Miron R. J. (2019). Macrophage behavior and interplay with gingival fibroblasts cultured on six commercially available titanium, zirconium, and titanium-zirconium dental implants. *Clin. Oral Investig.* 23 3219–3227. 10.1007/s00784-018-2736-z 30415441

[B101] Wang X. Freire Valls A. Schermann G. Shen Y. Moya I. M. Castro L. (2017). YAP/TAZ orchestrate VEGF signaling during developmental angiogenesis. *Dev. Cell* 42 462–478.e7. 10.1016/j.devcel.2017.08.002 28867486

[B102] Washio K. Tsutsumi Y. Tsumanuma Y. Yano K. Srithanyarat S. S. Takagi R. (2018). *In vivo* periodontium formation around titanium implants using periodontal ligament cell sheet. *Tissue Eng. Part A* 24 1273–1282. 10.1089/ten.tea.2017.0405 29495925

[B103] Wilson C. J. Clegg R. E. Leavesley D. I. Pearcy M. J. (2005). Mediation of biomaterial-cell interactions by adsorbed proteins: a review. *Tissue Eng.* 11 1–18. 10.1089/ten.2005.11.1 15738657

[B104] Wisdom C. Chen C. Yuca E. Zhou Y. Tamerler C. Snead M. L. (2019). Repeatedly applied peptide film kills bacteria on dental implants. *JOM* 71 1271–1280. 10.1007/s11837-019-03334-w 31178649 PMC6550465

[B105] Wu X. Ma Y. Su N. Shen J. Zhang H. Wang H. (2019). Lysophosphatidic acid: its role in bone cell biology and potential for use in bone regeneration. *Prostaglandins Other Lipid Mediat.* 143:106335. 10.1016/j.prostaglandins.2019.106335 31054330

[B106] Xiang L. Yu H. Zhang X. Wang B. Yuan Y. Zhang Q. (2018). The versatile hippo pathway in oral-maxillofacial development and bone remodeling. *Dev. Biol.* 440 53–63. 10.1016/j.ydbio.2018.05.017 29792855

[B107] Xiong J. Almeida M. O’Brien C. A. (2018). The YAP/TAZ transcriptional co-activators have opposing effects at different stages of osteoblast differentiation. *Bone* 112 1–9. 10.1016/j.bone.2018.04.001 29626544 PMC5970058

[B108] Yang B. Sun H. Xu X. Zhong H. Wu Y. Wang J. (2020). YAP1 inhibits the induction of TNF-α-stimulated bone-resorbing mediators by suppressing the NF-κB signaling pathway in MC3T3-E1 cells. *J. Cell. Physiol.* 235 4698–4708. 10.1002/jcp.29348 31642068

[B109] Yang W. Han W. Qin A. Wang Z. Xu J. Qian Y. (2018). The emerging role of Hippo signaling pathway in regulating osteoclast formation. *J. Cell. Physiol.* 233 4606–4617. 10.1002/jcp.26372 29219182

[B110] Yasuda D. Kobayashi D. Akahoshi N. Ohto-Nakanishi T. Yoshioka K. Takuwa Y. (2019). Lysophosphatidic acid-induced YAP/TAZ activation promotes developmental angiogenesis by repressing Notch ligand Dll4. *J. Clin. Invest.* 129 4332–4349. 10.1172/JCI121955 31335323 PMC6763231

[B111] Yu F.-X. Zhao B. Panupinthu N. Jewell J. L. Lian I. Wang L. H. (2012). Regulation of the Hippo-YAP pathway by G-protein-coupled receptor signaling. *Cell* 150 780–791. 10.1016/j.cell.2012.06.037 22863277 PMC3433174

[B112] Yung Y. C. Stoddard N. C. Chun J. (2014). LPA receptor signaling: pharmacology, physiology, and pathophysiology. *J. Lipid Res.* 55 1192–1214. 10.1194/jlr.R046458 24643338 PMC4076099

[B113] Zarb G. Albrektsson T. (1991). Osseointegration: a requiem for the periodontal ligament? *Int. J. Periodontics Restorative Dent.* 11 81–91.

[B114] Zhang Q. Guo Y. Yu H. Tang Y. Yuan Y. Jiang Y. (2019). Receptor activity-modifying protein 1 regulates the phenotypic expression of BMSCs via the Hippo/Yap pathway. *J. Cell. Physiol.* 234 13969–13976. 10.1002/jcp.28082 30618207 PMC13483059

[B115] Zhao L. Guan H. Song C. Wang Y. Liu C. Cai C. (2018). YAP1 is essential for osteoclastogenesis through a TEADs-dependent mechanism. *Bone* 110 177–186. 10.1016/j.bone.2018.01.035 29432919

[B116] Zhao X. Wang X. You Y. Wen D. Feng Z. Zhou Y. (2020). Nogo-B fosters HCC progression by enhancing Yap/Taz-mediated tumor-associated macrophages M2 polarization. *Exp. Cell Res.* 391:111979. 10.1016/j.yexcr.2020.111979 32246992

[B117] Zhou X. Li W. Wang S. Zhang P. Wang Q. Xiao J. (2019). YAP aggravates inflammatory bowel disease by regulating M1/M2 macrophage polarization and gut microbial homeostasis. *Cell Rep.* 27 1176–1189.e5. 10.1016/j.celrep.2019.03.028 31018132

[B118] Zhu W. Ming P. Qiu J. Shao S. Yu Y. Chen J. (2018). Effect of titanium ions on the Hippo/YAP signaling pathway in regulating biological behaviors of MC3T3-E1 osteoblasts: regulation of osteoblasts by Ti ions via Hippo/YAP. *J. Appl. Toxicol.* 38 824–833. 10.1002/jat.3590 29377205

[B119] Zizzi A. Aspriello S. D. Rubini C. Goteri G. (2011). Peri-implant diseases and host inflammatory response involving mast cells: a review. *Int. J. Immunopathol. Pharmacol.* 24 557–566. 10.1177/039463201102400302 21978688

[B120] Zohrabian V. M. Sonick M. Hwang D. Abrahams J. J. (2015). Dental implants. *Semin. Ultrasound CT MRI* 36 415–426. 10.1053/j.sult.2015.09.002 26589695

